# Contrasting Trends in Plant Diversity and Soil Carbon Mineralization Under Precipitation‐Driven Vegetation and Soil Carbon Dynamics in the Mongolian Plateau

**DOI:** 10.1002/ece3.71806

**Published:** 2025-07-15

**Authors:** Jia Mi, Fei Wang, Jing Shi, Qianju Wang, Haiyan Pang, Jianhao Yu, Dima Chen, Yongfei Bai

**Affiliations:** ^1^ Shanxi Key Laboratory for Ecological Restoration of Loess Plateau, Institute of Loess Plateau Shanxi University Taiyuan China; ^2^ Field Scientific Observation and Research Station of the Ministry of Education for Subalpine Grassland Ecosystem in Shanxi Ningwu China; ^3^ College of Environment and Resources Sciences Shanxi University Taiyuan China; ^4^ School of Ecology and Environment Inner Mongolia University Hohhot China; ^5^ State Key Laboratory of Vegetation and Environmental Change Institute of Botany, Chinese Academy of Sciences Beijing China; ^6^ College of Resources and Environment, University of Chinese Academy of Sciences Beijing China

**Keywords:** grassland ecosystem, impact pathway analysis, Mongolian plateau, plant species diversity, soil organic carbon mineralization

## Abstract

Climatic shifts critically regulate soil organic carbon (SOC) dynamics, biodiversity, and productivity in grasslands. However, the mechanisms linking abiotic/biotic factors to plant diversity–SOC mineralization synergies remain unclear. To study this mechanistic relationship, we established 12 sampling sites along an east–west precipitation gradient across the China‐Mongolia steppe. We measured plant diversity and biomass for both species and functional groups in 12 grassland sites. In the laboratory, we analyzed soil nutrients and microbial communities. We also measured the SOC mineralization potential using 28‐day incubations. The results indicated that: (1) Aboveground biomass (AGB) increased through two opposing strategies, enhancing or reducing plant diversity, with thresholds at Shannon–Wiener indices of 1.14 (arid west) and 2.19 (humid east). AGB shifts altered resource competition and microenvironments, directly impacting diversity. (2) Plant diversity was primarily regulated by soil pH, SOC, and mean annual temperature (MAT). (3) Perennial grasses dominated productivity, while perennial forbs drove diversity via niche complementarity. (4) Microbial biomass carbon (MBC) was the direct driver of SOC mineralization, modulated by mean annual precipitation (MAP) through SOC mediation. (5) SOC mediated contrasting ecosystem effects by suppressing plant diversity through pH‐driven nutrient limitations while simultaneously enhancing mineralization rates via stimulation of microbial decomposer activity. SOC properties and precipitation govern divergent changes in grassland diversity and carbon cycling. Strategic management of SOC pools, coupled with precipitation adaptation and biodiversity conservation, can enhance ecosystem resilience under climate change. This mechanistic framework advances understanding of grassland responses to global change.

## Graphical abstract

The relationship between aboveground biomass (AGB) and plant species diversity is complex, showing two strategies: higher and lower diversity with increased AGB. Soil organic carbon (SOC) exhibited contrasting effects on plant species diversity and SOC mineralization. The properties of SOC pools are important factors driving contrasting change in grassland plant species diversity and organic carbon mineralization in the typical steppe.

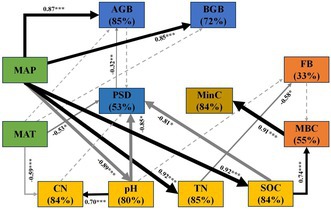

## 1 Introduction

Plant species diversity is a key feature of plant communities, encapsulating changes in species composition, distribution patterns, developmental stages, community stability, and interactions with geographic conditions (Hooper et al. 2012). Plant species diversity is influenced by the combined action from both biotic and abiotic factors (Pugnaire et al. 2019). Climate conditions, as one of the primary abiotic factors, crucially dominate the construction of the distribution pattern of plant diversity. Climatic conditions are a key factor influencing the distribution pattern of plant diversity. Studies have shown that warmer and wetter regions typically have higher species richness (Harrison et al. 2020), while warmer or drier conditions lead to significantly lower plant diversity (Klein et al. 2004; Bai et al. 2021). Beyond climatic influences, species diversity is also closely linked to soil environmental factors, including soil nutrient content (Chen et al. 2013), soil pH (Cambrollé et al. 2015), and the soil microbial community (Zak et al. 2003). The content of organic carbon in the soil, which is intimately connected to soil fertility, exerts a complex influence on species richness (van der Sande et al. 2018). Thus, it remains unclear whether higher soil organic matter content consistently supports greater species diversity. Soil organic matter modulates plant diversity through its regulation of nutrient acquisition competitiveness, structural porosity, and microbially‐mediated soil processes (Chen et al. 2013; Bai et al. 2021). Furthermore, the molecular structure and availability of soil organic carbon (SOC) significantly affect its stability (Xiao et al. 2018).

SOC mineralization is a complex microbial‐driven process directly tied to carbon inputs and outputs in the soil (Zhang, Liu, et al. 2022). Climatic factors and soil physicochemical properties jointly influence SOC mineralization by altering its content and composition. Conversely, microbial decomposition of plant litter increases the SOC content and thus the carbon mineralization rate (Bai et al. 2024). Temperature and moisture are key environmental factors regulating SOC mineralization, primarily reflected in their significant effects on microbial activity. Laboratory incubation experiments demonstrate that the rate of SOC mineralization increases with rising temperature and soil moisture (Curtin et al. 2012). Contrarily, experimental warming has been shown to negatively impact SOC mineralization (Zhou et al. 2012). Additionally, plant community composition can influence both SOC mineralization and microbial community structure (Shu et al. 2023). The increase in soil particle size also significantly increases the rate and accumulation of SOC mineralization (Cheng et al. 2024). Exploring SOC mineralization and its drivers offers deeper insights into the physicochemical and biological processes governing ecosystem carbon cycling.

Semi‐arid grasslands in northern China are representative of the Eurasian temperate grassland biome. Sand encroachment, salinization, and desertification of semi‐arid grassland ecosystems have intensified due to both natural processes and anthropogenic activities (Bardgett et al. 2021). Annual and biennial herbs diversity is more responsive to climate change than the diversity of perennial species (Thuiller et al. 2005). Rising temperatures in the Inner Mongolian grasslands have significantly suppressed plant diversity and biomass (Bai et al. 2021). Plant species diversity within communities is closely associated with the primary factors influenced by soil microbial composition, such as grassland productivity, carbon allocation, and soil nutrient cycling (Wardle et al. 2004; Xing et al. 2019). Changes in plant species diversity can also modify the soil microbial composition in microbial communities (Zak et al. 2003). Soil microbial community is the key regulatory factor for CO_2_ emissions out of soil carbon mineralization (Mi et al. 2015). Plant diversity could drive SOC storage through the chemical composition of plant organic matter, and climatic conditions strengthen this effect (Spohn et al. 2023). Climate change alters plant–soil feedbacks by modifying species traits and plant‐derived inputs, thereby influencing ecosystem functions such as soil organic carbon decomposition (Pugnaire et al. 2019). To conserve and sustainably manage semi‐arid grasslands, it is critical to understand how plant diversity shapes carbon storage across varying environmental conditions.

Prior research has concentrated on the direct drivers of SOC decomposition, such as microbial communities and SOC physicochemical properties, yet neglected the indirect regulatory mechanisms mediated by plant diversity (Mi et al. 2015). This study integrated multidimensional factors such as climatic conditions, plant community features, soil physicochemical properties and nutrient content, and soil microbial composition to systematically explore the complex pathways and key driving mechanisms underlying plant species diversity and soil carbon dynamics in Eurasia steppe. Our objectives were to: (1) elucidate the mechanisms linking plant diversity to SOC storage, with a focus on SOC accumulation and mineralization as the dominant driver; (2) investigate how climatic conditions modulate the relationship between plant diversity and SOC dynamics, including its stabilization or mineralization. According to the research content, we tested two hypotheses: firstly, increasing grassland biomass exhibits a nonlinear relationship with plant diversity, governed by precipitation‐driven thresholds; secondly, SOC drives divergent ecosystem outcomes by reducing plant diversity via pH‐dependent nutrient limitation while accelerating mineralization through microbial characteristics activation, with precipitation modulating the SOC to microbial characteristics pathway.

## 2 Materials and Methods

### 2.1 Study Area

The research was carried out in the steppe of Inner Mongolia, located in northern China, and serves as a prime example of the Eurasian steppe concerning its climate, soil, and vegetation (Bai et al. 2008). The geographical coordinates of the study area range from 39°01′ to 49°32′ N and 101°37′ to 120°02′ E, with elevations between 653 m and 1478 m. Mean annual temperature fluctuates from 2°C to 8°C, with January recording the lowest monthly averages (−9°C to −26°C) and July the highest (19°C–24°C). The region experiences mean annual precipitation varying from 100 mm to 380 mm, with approximately 80% of it falling during the growing season (May to August), aligning with peak temperatures (Bai et al. 2008). The soil types found here include chestnut, calcareous brown, and desert soils (IUSS Working Group WRB 2022). A total of twelve sites across the east–west gradient of decreasing precipitation were selected to represent four types of plant communities: meadow steppe, typical steppe, desert steppe, and steppe desert. The eastern meadow steppe is characterized by dominant species such as *Leymus chinensis*, *Stipa baicalensis*, and *Filifolium sibiricum* . In contrast, the central typical steppe features *Leymus chinensis*, *Stipa grandis* , and *Artemisia frigida* as predominant species. The desert steppe areas are primarily composed of *Stipa klemenzii* and *Allium polyrhizum*, while the western steppe desert areas are characterized by *Salsola passerina*, *Hololachna songarica*, and *Nitraria tangutorum* (Mi et al. 2015).

### 2.2 Study Site Setup and Plant–Soil Sampling Methodology

This study involved fieldwork and sampling in August 2012. Twelve sites were selected along an east–west belt transect (Mi et al. 2015). A sampling area of 100 m × 100 m was set up at each sample point, a sample line was laid out along the diagonal, and 10 sample squares of 1 m × 1 m were uniformly distributed on the sample line, with a spacing of 10 m between the squares to ensure uniformity and representativeness of the spatial distribution of the samples. Various measurements were conducted within these quadrats, focusing on plant species composition, both aboveground and belowground biomass, soil microbial communities, and the physical and chemical characteristics of the soil.

The number of plant species and their respective abundances was documented in each sampling plot. The plant species were classified into four categories according to their life forms: annual and biennial herbs (AB), perennial grasses (PG), perennial forbs (PF), and shrubs or subshrubs plants (SHS). Counts and biomass measurements were recorded according to plant functional groups, and by calculating the proportion of the individual count or biomass of each species relative to the total in the biological community, the relative abundance and relative biomass of the species can be determined.

Plant specimens within the sample plots were clipped at ground level. After collecting the aboveground biomass, three soil cores were randomly collected from five sample plots at depths of 0–5 cm, 5–10 cm, and 10–20 cm using a 7 cm diameter soil core drill. The three soil cores from each sample plot were mixed in situ to form a composite sample, which was then placed in a root bag at each sampling site. Soil in the root bags was washed with running water. Roots were separated using a 1 mm sieve, eliminating dead roots and other residues. The samples of stem, leaf, and root were dried in an oven at 65°C for at least 48 h until they reached a constant weight, after which they were weighed. The dry weight of biomass was divided into aboveground biomass (AGB) and belowground biomass (BGB).

Using a soil core drilling with a 5 cm diameter, soil samples were taken. The cores from three cores were merged to create a composite sample, which was subsequently sieved through a 2 mm mesh, air‐dried, and cleaned of gravel and other impurities prior to analyzing the physicochemical properties. The pH of the soil was determined with a potentiometric method, using a 2.5:1 liquid‐to‐soil ratio and the FE28 pH meter (Mettler Toledo, Switzerland). Total carbon (TC) and total nitrogen (TN) content (g/kg of dry soil) were measured by a CHON elemental analyzer (Elementar VARIO EL III, Hanau, Germany). Inorganic carbon (IC) was determined using a calcium carbonate analyzer (Eijkelkamp, Giesbeek, Netherlands). SOC was then calculated by subtracting IC from TC. The carbon‐to‐nitrogen ratio (C/N) was obtained by dividing SOC by TN.

Microbial biomass carbon (MBC) was assessed through the chloroform fumigation‐K_2_SO_4_ extraction technique (Vance et al. 1987). The analysis of the soil microbial community was conducted using the phospholipid fatty acid (PLFA) method (Buyer and Sasser 2012). The soil samples used for PLFA analysis were subsamples from previous soil cores and were stored in freezers at −20°C after sampling. The fungi‐to‐bacteria ratio (F:B) was obtained from PLFA data.

### 2.3 Meteorological Data and Soil Organic Carbon Mineralization

Meteorological data were sourced from nearby meteorological stations at the sampling locations within the study area. Daily precipitation and temperature readings for each sampling site were derived and processed using O‐Kriging spatial interpolation within the Geographic Information System (ArcGIS). Interpolation accuracy was verified against station records (*R* ^2^ > 0.85, RMSE < 15% of mean values). Geospatial analysis and mapping were conducted using ArcGIS 10.3 (ESRI 2014. ArcGIS Desktop: Release 10.3. Redlands, CA: Environmental Systems Research Institute). The digital elevation model (DEM) data has a resolution of 30 × 30 m, employs the Lambert projection, and uses the WGS‐84 geodetic coordinate system with the Krasovsky ellipsoid. The measured data in 2010 (*n* = 147) were used to test the interpolation value (Chen et al. 2013). We used these data to calculate the mean annual temperature (MAT) and the mean annual precipitation (MAP). Data on SOC mineralization and carbon dioxide accumulation from incubated soils under conditions of 25°C and 60% water‐filled pore space (WFPS) across the 12 sampling sites were retrieved from our prior research for additional analysis (Mi et al. 2015). All soil samples used for routine physicochemical analyses and incubation experiments originate from the same batch of air‐dried soil subsamples (sieved < 2 mm), ensuring direct comparability between analyses (Powlson et al. 1987).

### 2.4 Statistic Analysis

Plant species diversity (PSD) is frequently assessed using the Simpson diversity index, Shannon–Wiener diversity index, and Margalef richness index. The Shannon–Wiener index was used to assess species richness and evenness, the Simpson index to measure community dominance, and the Margalef index to characterize species richness in order to comprehensively analyze plant community diversity characteristics (Loreau and Hector 2001). The formulas for each index were as follows:

$$\text{Simpson index}:D=1-\sum {P_{i}}^{2}$$

$$\text{Shannon}–\text{Wiener diversity index}:H^{\prime }=-\sum P_{i}\ln P_{i}$$

$$\text{Margalef richness index}:R=\left(S-1\right)/\ln N$$

where *S* denotes the total number of species within the community; *N* represents the overall count of individuals across all species in the community; *N*_*i*_ indicates the number of individuals for each species, and *P*_*i*_ = *N*_*i*_/*N*. The variable *i* signifies the relative density of plant species, calculated as the number of individuals of a particular species divided by the total number of individuals of all species.

Univariate regression was employed to quantify relationships between continuous environmental predictors and core response variables (biomass, SOC accumulation, and mineralizable carbon); and relationships between diversity indices (Simpson, Shannon–Wiener, Margalef) and biomass by using SPSS version 23.0 (IBM Corp., Armonk, NY, USA). This would help verify whether variables meet the fundamental assumptions of SEM modeling and provide theoretical foundations for constructing structural equation modeling (SEM) pathways. The regression equation was fitted by selecting the function that yielded the highest coefficient of determination (*R* ^2^). Mantel test and Pearson's test were used to investigate the correlation between diversity indices as well as SOC mineralization accumulation and environmental variables through using R software version 4.1.2 (Lucent Technologies, Jasmine Mountain, New Jersey, USA) and the R packages (‘stats’ R‐package for the Pearson test, ‘ggplot2’ and ‘linkET’ R‐package for the Mantel test and data visualization). SEM is used to construct a hypothesis model between variables, including climate factors (MAP, MAT), plant community traits (AGB, BGB, PSD), soil physicochemical properties (pH, C:N, TN, SOC), soil microbial characteristics (MBC, F:B), cumulative mineralization of SOC (MinC). The PSD was represented by the Shannon–Wiener diversity index in the model. The SEM analyses were performed using Amos version 17.0.2 (Amos Development Corporation, Chicago, IL, USA), applying chi‐square (*χ* ^2^) tests, comparative fit indices (CFI), root mean square error of approximation (RMSEA), and goodness of fit index (GFI) to evaluate model performance (MacCallum and Austin 2000).

## 3 Results

### 3.1 Relationship Between Diversity Index and Climatic Conditions

**FIGURE 1:**
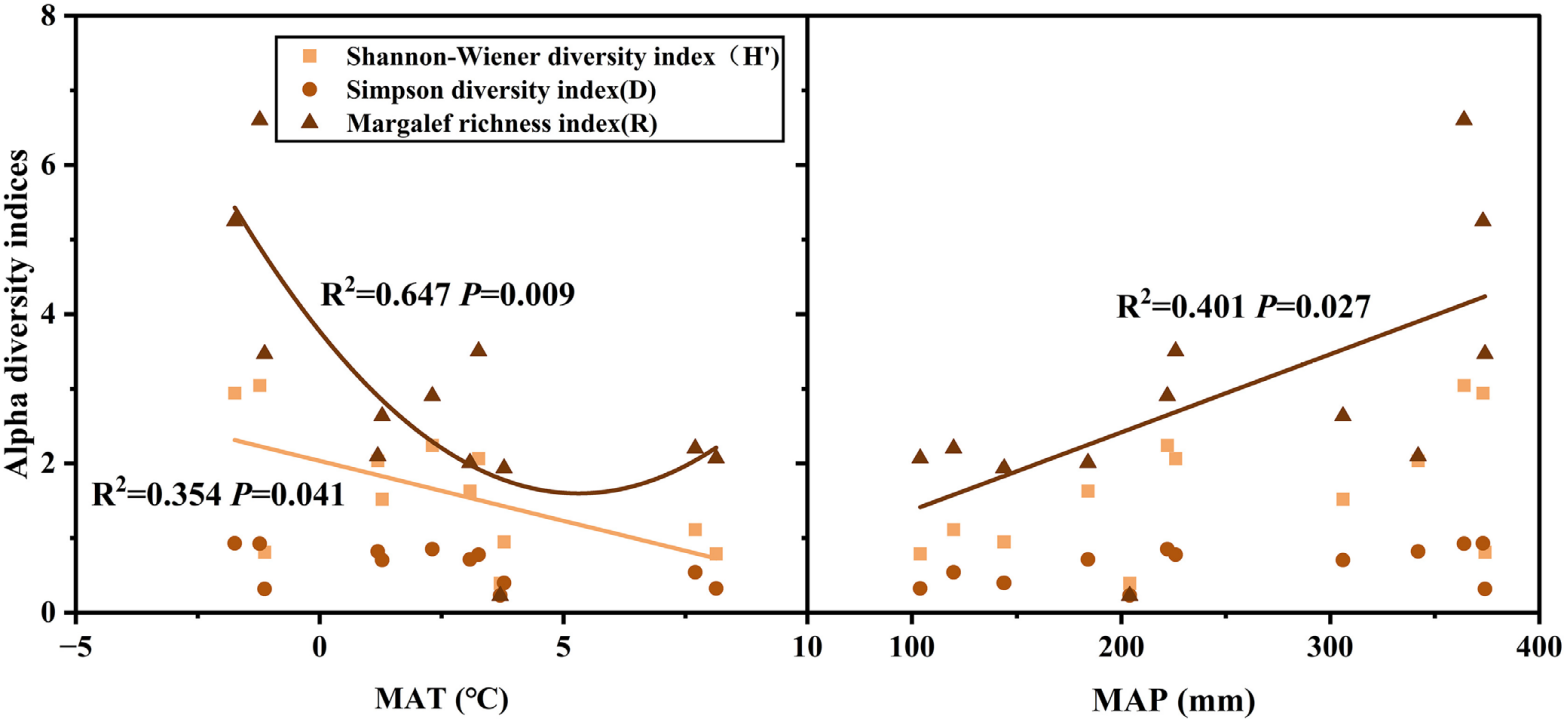
Relationship between MAT and MAP and plant species diversity index in the study area (Only statistically significant regression relationships are shown (*p* < 0.05), non‐significant relationships were not shown in the figure, applies to all figures).

There are differences in the responses of various plant diversity indices (Shannon–Wiener index, Margalef richness index, Simpson index) to climatic factors (Figure 1). The Shannon–Wiener index showed a decreasing trend with increasing MAT, while the Margalef richness index exhibited a significant “*U*‐shaped” relationship with MAT (*p* < 0.05), indicating that both increases and decreases in MAT were accompanied by increases in the Margalef richness index. Meanwhile, the Margalef richness index increased with higher MAP. In contrast, the Simpson index showed no significant association with any of the climatic factors.

### 3.2 Relationship Between Climatic Conditions and Community Biomass

**FIGURE 2:**
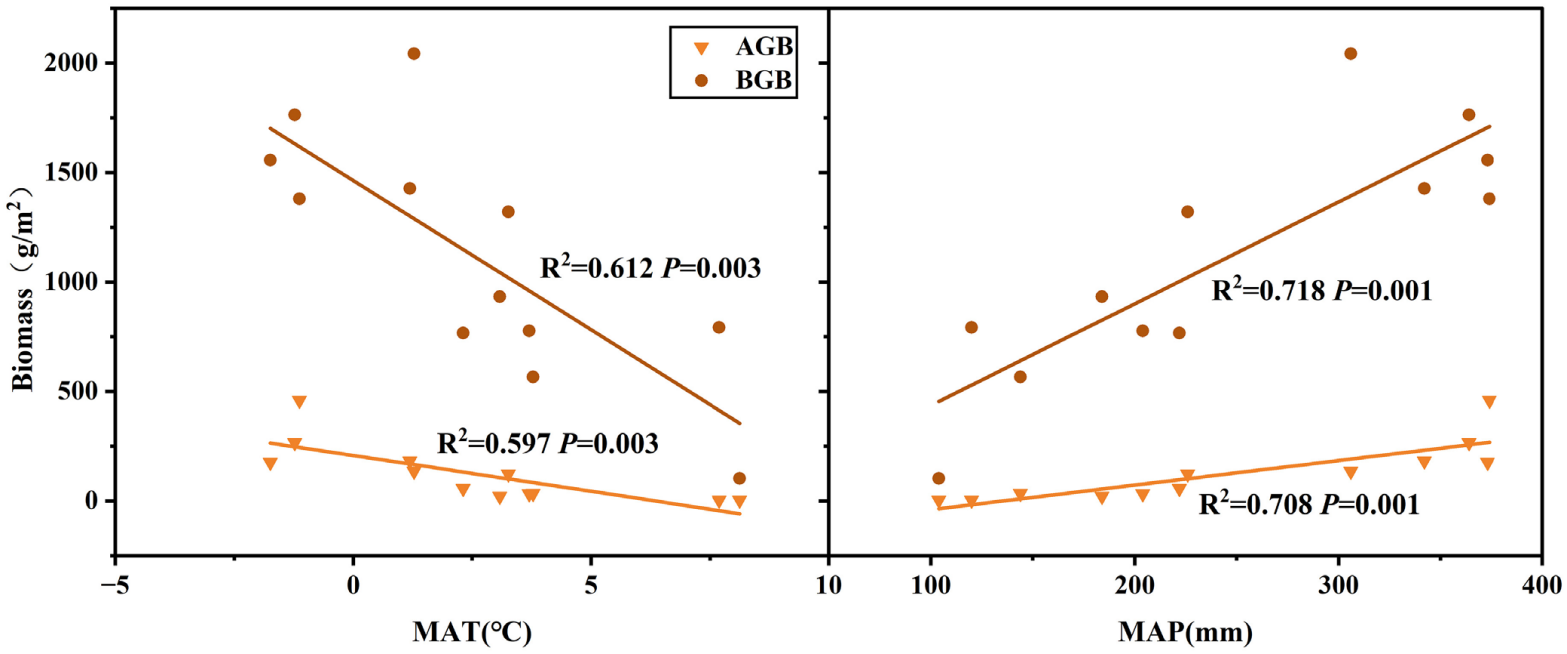
Relationship between MAT and MAP and aboveground and belowground biomass of communities in the study area.

Both AGB and BGB significantly decreased with MAT but increased with MAP (Figure 2). BGB changes were greater than those for the AGB, both with MAT and MAP. As shown in Figure S1, the relative biomass of perennial grass plants in the community showed significant linear regression relationships with both MAT and MAP (*p* < 0.05). With decreasing MAT or increasing MAP, the relative biomass of perennial grass plants in the community gradually increased. The relative biomass of shrub or subshrubs plants gradually increased with rising MAT, while showing a significant quadratic curve regression relationship with MAP (*p* < 0.05). The increase in relative biomass of shrub or subshrubs plants was accompanied by either increases or decreases in MAP.

### 3.3 Relationship Between Diversity Index and Community Biomass

**FIGURE 3:**
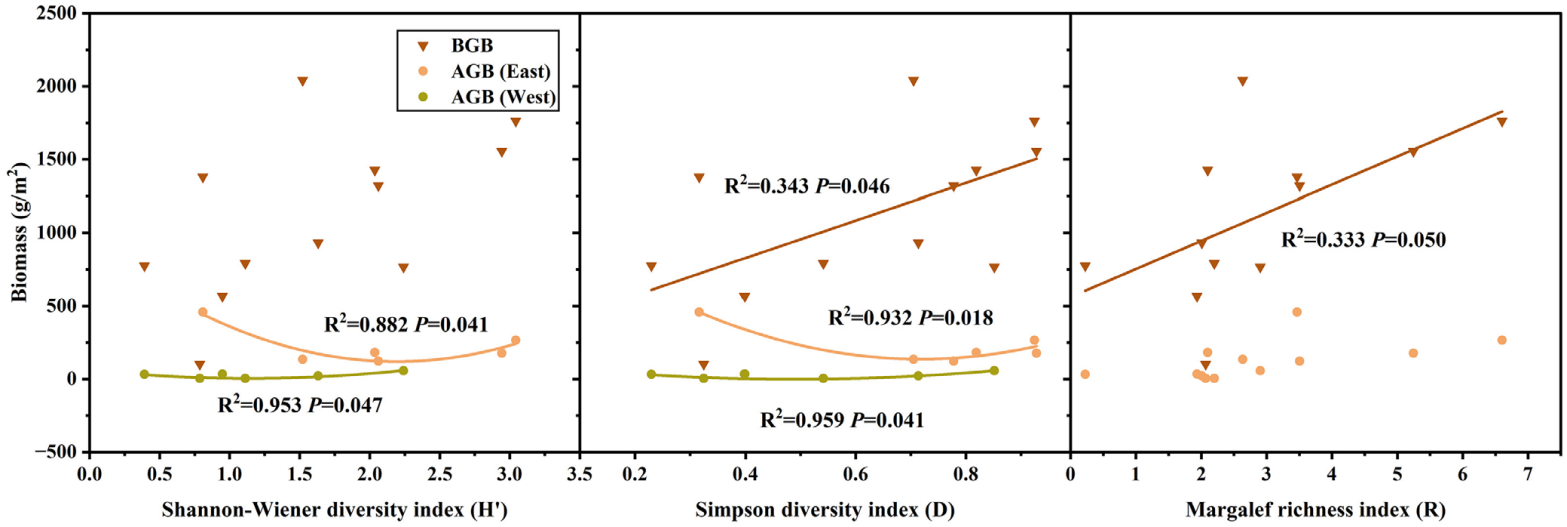
Relationship between biodiversity indices and above and belowground biomass of the communities. AGB (West): Above ground biomass in the western part of the study area (green dot and line: 6 sites in the western); AGB (East): Aboveground biomass in the eastern part of the study area (yellow dot and line: 6 sites in the eastern); BGB, belowground biomass (brown triangle and line: 12 sites).

A notable quadratic regression relationship (*p* < 0.05) was identified between AGB and both the Shannon–Wiener and Simpson indices; this quadratic regression suggests higher AGB values were found in plant communities with the highest and lowest species diversity (Figure 3). The analysis revealed that AGB reached a minimum of 5.22 g/m^2^ in the six western sites at a Shannon–Wiener index of 1.14, while in the eastern sites, it exceeded 120.06 g/m^2^ at a diversity index of 2.19. For the Simpson index, the lowest AGB in the west region was 0.49, compared to 136.82 g/m^2^ in the east at an index of 0.72. There was no significant correlation or regression observed between AGB and the Margalef richness index. However, a significant positive linear regression relationship (*p* < 0.05) was found between the Margalef richness index and Simpson's index with BGB. Additionally, no significant correlation or regression relationship was identified between BGB and the Shannon–Wiener index.

### 3.4 Analysis of Influencing Factors on Relative Biomass of Different Plant Functional Groups

**FIGURE 4:**
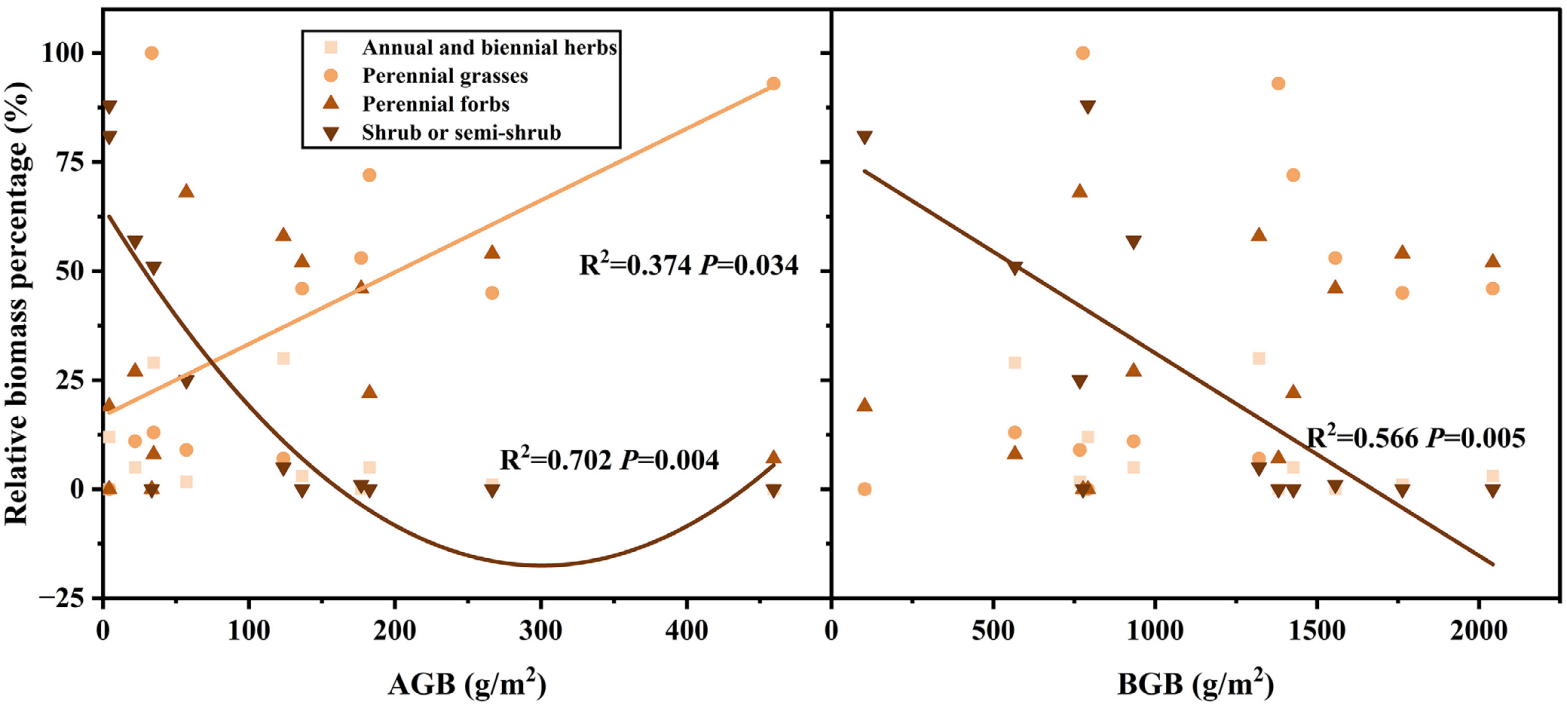
Aboveground and belowground biomass of communities with the relative biomass of different plant functional groups.

The relative biomass of shrubs or subshrubs plants showed a significant quadratic regression relationship (*p* < 0.05) with AGB (Figure 4). The relative biomass of shrubs or subshrubs was highest in plant communities with the lowest or highest AGB values, but decreased in sites where shrub species had higher AGB. A significant linear regression relationship (*p* < 0.05) was found between the relative biomass of shrub or half‐shrub species and BGB. With increased BGB, the relative biomass of shrub or half‐shrub plant functional groups gradually decreased. No significant correlation was found between perennial forbs, perennial grasses, annual or biennial herbs, and BGB. However, a significant linear regression relationship (*p* < 0.05) was identified between the relative biomass of perennial grasses and AGB, indicating that the relative biomass of perennial grasses increased steadily as AGB rose.

Comparative analysis of biomass percentages by plant family across sampling sites revealed (Figure S5): steppe desert was dominated by Chenopodiaceae (38%) and Gramineae (33.3%); desert steppe transitioned to co‐dominance by Chenopodiaceae (33.7%), Compositae (16.3%) and Leguminosae (17.3%); typical steppe showed Gramineae (43%) as the dominant family with accompanying Liliaceae (26%) and minor Chenopodiaceae (10.7%); while meadow steppe was markedly dominated by Gramineae (64%). Therefore, at the large scale, the dominant species in Inner Mongolia grasslands shifted from Chenopodiaceae to Gramineae along the west–east temperature and precipitation gradient.

**FIGURE 5:**
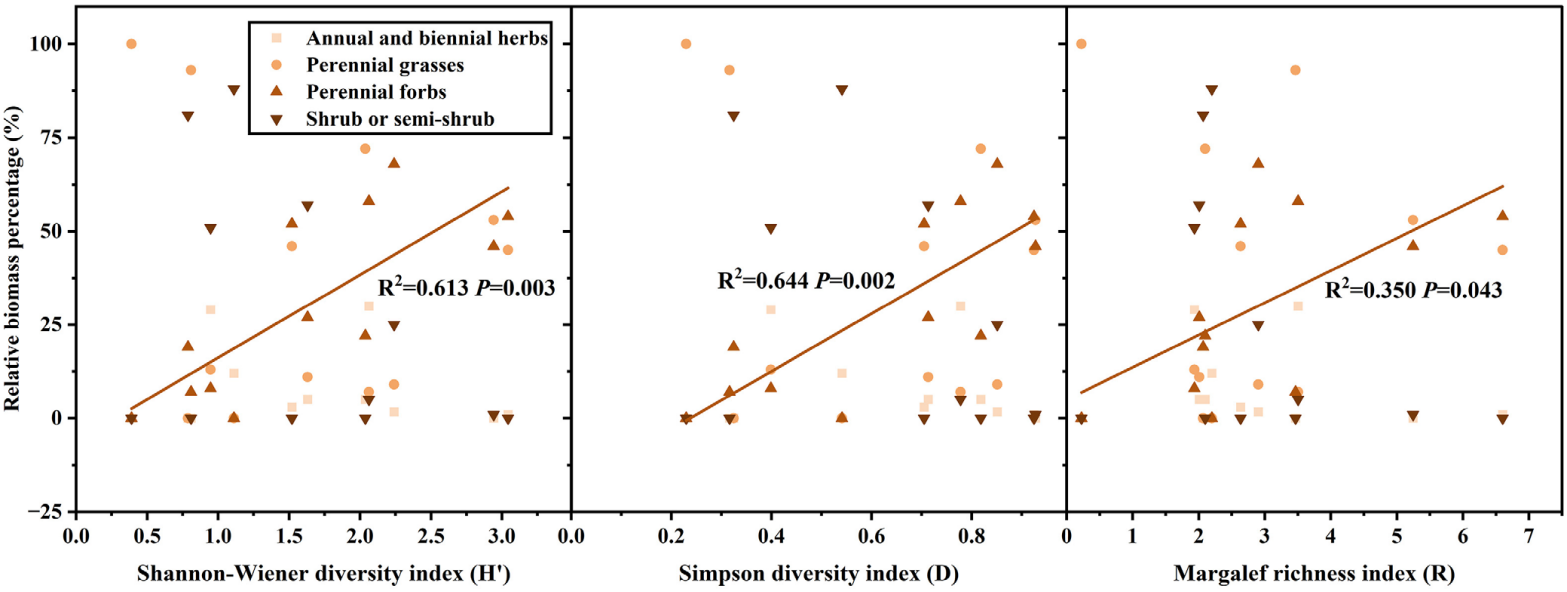
Representation of the relative biomass of plant functional groups in relation to diversity indices along the belt transect.

The relative biomass of plant functional groups showed significant variation in its correlation with biodiversity indices in the grassland community (Figure 5). Only the relative biomass of perennial forbs demonstrated a significant positive linear regression (*p* < 0.05) with the Shannon–Wiener index, Simpson index, and Margalef richness index.

**FIGURE 6:**
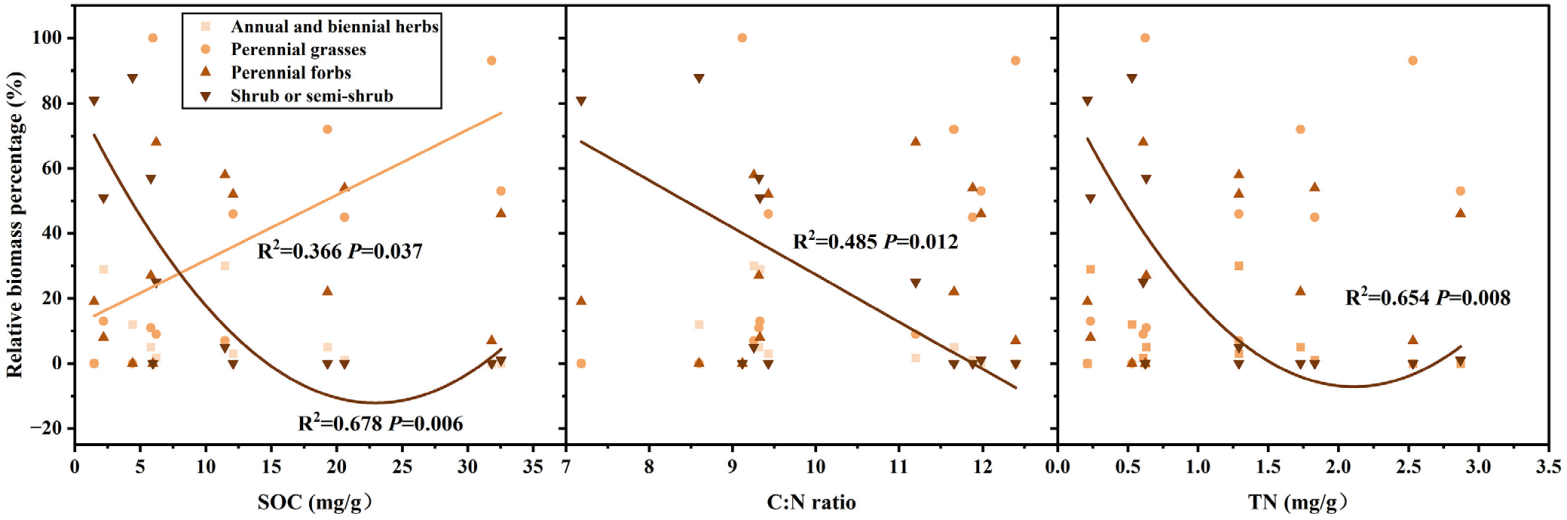
Influences of soil organic carbon (SOC), total nitrogen (TN), and carbon‐to‐nitrogen ratio (C:N) on the relative biomass of various plant functional groups.

The relative biomass of shrub or subshrub species showed a significant quadratic regression with SOC and TN (*p* < 0.05) (Figure 6), initially decreasing and then increasing as SOC and TN levels rose. Additionally, there was a significant linear regression (*p* < 0.05) between the relative biomass of both shrub or subshrub and the C:N, with biomass decreasing as C:N increased. A significant linear relationship (*p* < 0.05) was also observed between perennial grasses and SOC. However, no significant linear regression was found between the relative biomass of perennial grasses and either C:N or TN. In contrast, no significant correlations were detected between the relative biomass of perennial forbs and SOC, C:N, or TN.

### 3.5 Analysis of Potential Links and Pathways Between Factors

Analysis of factors affecting SOC mineralization accumulation after 28 days of incubation showed that with increasing MAT, SOC mineralization accumulation in both 0–5 and 5–10 cm soil layers gradually decreased, while MAT had no significant effect on the 10–20 cm layer. In contrast, SOC mineralization accumulation in all three soil layers increased with increasing MAP (Figure S2). Regarding soil properties, SOC mineralization accumulation in both 0–5 and 5–10 cm layers increased with increasing SOC and TN content, while SOC and TN showed no significant effect on the 10–20 cm layer (Figure S3). Furthermore, SOC mineralization accumulation in all three soil layers (0–5, 5–10, and 10–20 cm) showed an increasing trend with increasing MBC and fungal/bacterial biomass (Figure S4).

The results of Mantle's test (Figure S6) showed that perennial herbaceous plants had a significant effect (*p* < 0.05) on plant diversity index, while MAT, shrub or subshrub relative biomass, MAP, MBC, fungi, bacterial counts, BGB, SOC, TN, and soil pH values all had significant (*p* < 0.05) effects on organic carbon mineralization accumulation. Among them, the effects of MAP, SOC, MBC, and TN were the most significant.

**FIGURE 7:**
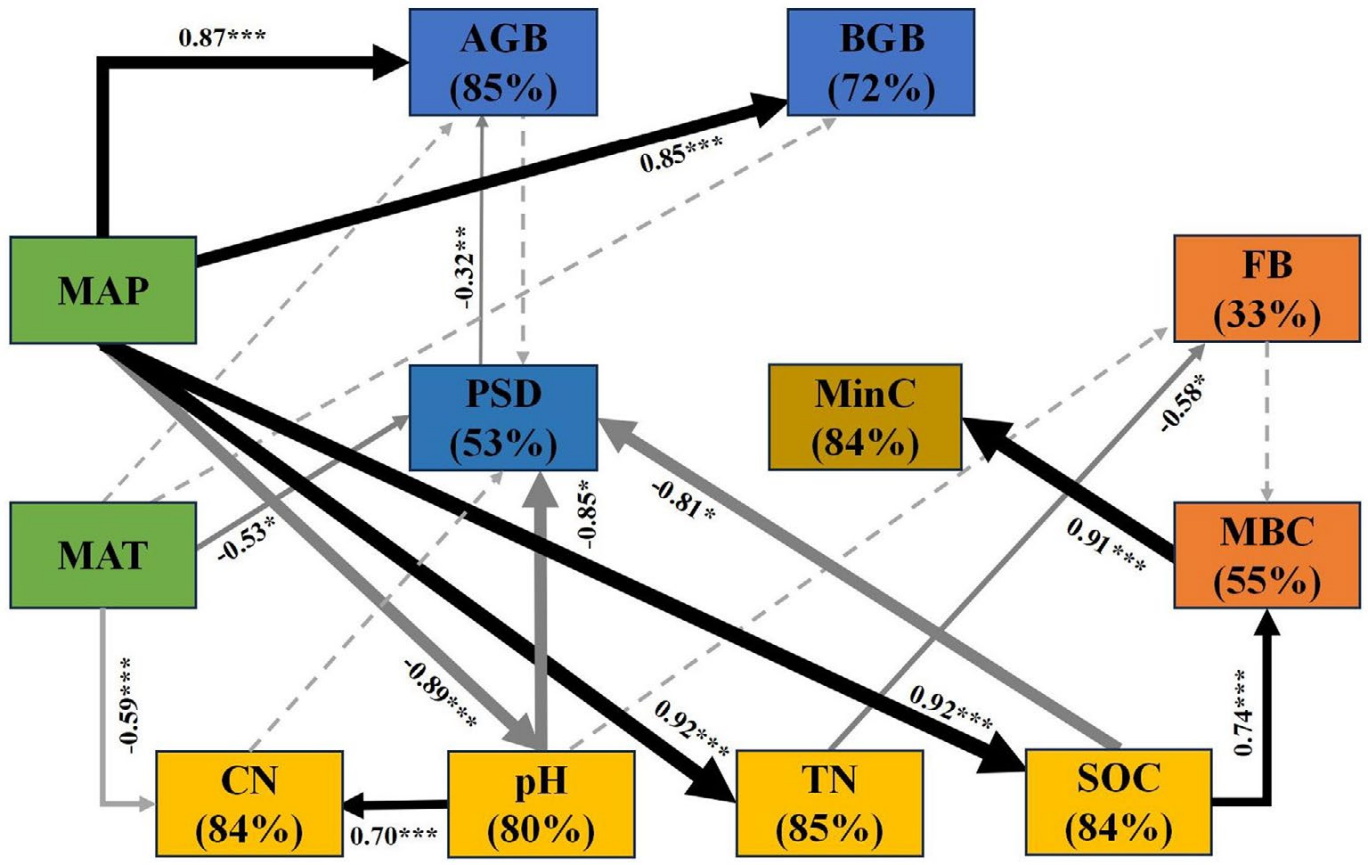
Structural equation modeling (SEM) showing causal relationships between climate, vegetation, and soil factors on plant diversity and SOC mineralization. Black arrows indicate positive effect, while gray arrows represent negative effect. The values represented by solid arrows denote standardized path coefficients (β), with significance indicated as ***p < 0.001, **p < 0.01, and *p < 0.05. Non‐significant pathways, represented by dashed arrows, were included to achieve the most parsimonious model. The values within brackets represent the proportion of variation accounted for by the relationships with other variables. MAP, mean annual precipitation; MAT, mean annual air temperature; pH, soil pH value; SOC, soil organic carbon; MBC, soil microbial biomass carbon; PSD, plant species diversity (Shannon–Wiener diversity index was used in this model); C:N, soil carbon to nitrogen ratio; TN, total soil nitrogen; AGB, aboveground biomass; BGB, belowground biomass; F:B, soil fungi and bacteria ratio; MinC: Cumulative mineralization of SOC.

The SEM exhibited a good fit to the data (*χ* ^2^ = 480.504, df = 52, *p* = 0.209) (Figure 7). The key findings included as follows. Regulation of plant diversity: MAT, soil pH, and SOC had significant negative effects on the Shannon–Wiener diversity index (standardized path coefficients (β) were −0.32, −0.41 and −0.29, respectively), which together accounted for 53% of the variation. Regulation of AGB: MAP directly positively regulates AGB (β = +0.68), while the diversity index has a negative effect on AGB (β = −0.54), jointly explaining 85% of AGB variance. Soil dynamics: MAP directly promoted the accumulation of BGB (β = +0.85, 72% variation), SOC (β = +0.92, 84% variance) and TN (β = +0.88, 85% variance). TN suppressed F:B (β = −0.57, 33% variance), while SOC elevated MBC (β = +0.74, 55% variance), driving 84% of MinC variance. Reciprocal inhibition: MAP reduced soil pH (β = −0.89, 80% variance). MAT and pH jointly lowered the soil C:N (β = −0.67/−0.71, 84% variance). The model reveals a cascade effect of climate‐soil‐biomass synergistic regulation of carbon and nitrogen cycles in semi‐arid grassland ecosystems (Figure 7).

The results in Figure S8 demonstrate that MAT exerts a direct negative regulatory effect on the relative biomass of perennial forb plants. MAP influences the ecosystem through dual pathways: on one hand, it directly regulates the relative biomass of perennial grass plants, thereby affecting AGB; on the other hand, it indirectly affects the biomass of perennial forbs by regulating SOC, consequently influencing plant species diversity indices. MAT and SOC collectively explain 63% of the total variance in relative biomass of perennial forb plants, while MAP accounts for 44% of the total variance in relative biomass of perennial grass plants.

## 4 Discussion

### 4.1 Multiple Drivers of Plant Species Diversity

Plant diversity, which underpins the structure and function of ecosystems, is profoundly affected and increasingly threatened by climate change. A reduction or loss of plant diversity inevitably impacts ecosystem structure and functional stability, resulting in significant ecological, economic, and social consequences. The response of plant diversity to climate change is governed by the dynamic equilibrium between temperature and moisture, as well as the subsequent changes in the soil environment (Pugnaire et al. 2019). In this study, soil pH, SOC, and MAT are identified as three main drivers influencing plant diversity (Figure 7). The study area is extensive, with complex soil conditions. Soil pH regulates plant diversity through three interconnected pathways: nutrient‐toxicity tradeoffs, microbiome‐mediated feedbacks, and soil structural constraints (Cambrollé et al. 2015; Liu et al. 2022; Bolan et al. 2023). Changes in environmental temperature have a significant impact on the hydrothermal dynamics within ecosystems, not only significantly modifying species composition and community structure but also leading to a declining trend in plant diversity (both richness and evenness) along gradients of increasing aridity or latitude (Soliveres et al. 2014). In the semi‐arid temperate steppe zone, increasing temperatures are accompanied by soil drought. Drought may interact synergistically with soil pH, causing adverse physiological effects (e.g., osmotic stress) on species diversity (Palpurina et al. 2017). In this study area, plant species diversity exhibited a linear negative relationship with MAT (Figures 1 and 7).

SOC content was positively correlated with the Shannon index across 84 sites for grassland ecosystems at the global scale (Spohn et al. 2023). Contrasting with the findings of this study, the plant species diversity index demonstrated a negative relationship with SOC (Figure 7). An increase in MAP enhances SOC accumulation, which in turn reduces plant species diversity within the community by affecting perennial forb species (Figure S8). This phenomenon may be attributed to the positive correlation between higher SOC levels and increased biomass in the perennial grasses functional group (Figure 6). Such conditions constrain the growth of perennial forbs functional groups, consequently leading to reduced overall plant species diversity. Conversely, individual species (*Polygonum criopolitanum* or *Carex thunbergii* ) within a community may be more responsive and competitive for soil resources than others, potentially dominating in resource‐poor environments and reducing plant diversity (Huangfu et al. 2022). MAP and MAT exerted divergent effects on soil C:N ratios (Figure S7), yet C:N ratios showed no significant direct association with plant diversity indices. The loss of plant diversity in our experiments was accompanied by higher soil C:N (nitrogen deficiency). Soil microorganisms were more likely to have a greater impact on SOC degradation than its stabilization under low soil C:N conditions (Creme et al. 2017). This likely reflects regional‐scale characteristics of azonal meadow grasslands, which typically maintain adequate moisture but nitrogen‐limited soils. The influence of soil nutrient changes, in conjunction with precipitation gradients and variations in soil pH, on grassland plant species is complex (Figure 7). Overall, both pH and SOC regulated by MAP impact the distribution patterns of plant species. Additionally, soil nutrient availability and stoichiometry may indirectly influence plant species diversity by affecting primary productivity (Chen et al. 2013).

Climatic conditions significantly influence plant species diversity (Rey et al. 2005). In this study, the Shannon–Wiener index decreased with increasing MAT (Figure 1), indicating a negative correlation between MAT and the diversity index (Figure 7). On the one hand, temperature changes may alter species distribution patterns, as species often adapt to climate change by migrating to new habitats or developing new biological traits, potentially altering plant diversity (Lawlor et al. 2024). Conversely, rising temperatures may alter species interactions by changing resource requirements (García et al. 2018). Here, we emphasize the mechanistic basis of these opposing trends in areas where MAP increases offset MAT‐driven aridification; diversity could remain stable or even increase. This would help identify MAT/MAP tipping points for key functional groups.

Additionally, in this study, the diversity index was significantly correlated with the relative biomass of the perennial forbs functional group, whose biomass increased with diversity (Figure 5). For example, Compositae and Chenopodiaceae possibly played a key role in maintaining grassland diversity (Figure S5), indicating that the plant species diversity of this community was primarily driven by the dominance of the perennial forbs functional group. The high contribution of perennial forbs to species diversity may stem from the varying abilities of different functional groups to influence diversity in different grassland types due to their unique characteristics (Li et al. 2021). These traits, including competitive dominance, ecological adaptability, and mixed reproductive strategies, enable perennial forbs to act as ecosystem engineers that indirectly maintain plant diversity. Their broad niche space (e.g., deep roots) creates microhabitat heterogeneity, allowing coexistence of species with divergent resource requirements (Huang et al. 2025). By stabilizing soils and moderating microclimates, they facilitate colonization by stress‐intolerant species (Rojas‐Botero et al. 2023). While suppressing ruderal competitors, their patch dominance generates light/resource gaps that enable fugitive species persistence (Zhang, Su, et al. 2022). Thus, rather than merely sustaining themselves, this functional group structures diversity through context‐dependent competition–facilitation balances, a mechanism critical for grassland resilience.

### 4.2 Trade‐Off of Plant Diversity and Grassland Productivity

Our study showed that higher MAT reduced both AGB and BGB, while increased MAP improved biomass (Figure 2). Biomass was mainly controlled by MAP (Figure 7), highlighting moisture as the key driver in this grassland system. Biomass strongly correlated with perennial grasses and shrubs (Figures S1 and S2). However, shrubs or subshrubs were rare in eastern Inner Mongolia, meaning perennial grasses dominated productivity (Li et al. 2021). These perennial grasses also sustained AGB, and perennial grass species dominated sites with lower MAT and high MAP. Both temperature and precipitation shaped plant group distribution (Chen et al. 2015).

The relationship between plant community productivity and diversity has been extensively investigated and remains a central focus in ecological research (Rajaniemi 2003). Several forms of relationships between species diversity and productivity have been observed, including positive, negative, and single‐peak curves, with the single‐peak relationship being the most common (Fraser et al. 2015). In this study, the results demonstrated that increased productivity was associated with a decline in diversity (Figure 7). However, the process is complex and nonlinear, and no significant relationship was detected across the 12 sample points. When analyzing the six wetter eastern points and the six drier western points separately, a potential pattern emerged (Figure 3). Whether in the relatively arid western region (Shannon–Wiener index 1.14, Simpson index 0.49) or the more humid eastern region (Shannon–Wiener index 2.19, Simpson index 0.72), the increase in AGB was always accompanied by two opposite trends in diversity indices (Figure 3). This phenomenon suggests that increased grassland community productivity at the threshold point was achieved through two distinct strategies: either elevating or reducing species diversity.

Some studies often found hump‐shaped patterns (most productive at moderate diversity) (Fraser et al. 2015); our data showed productivity rising as diversity declined (Figure 7). However, this trend was inconsistent across all 12 sites. When split into wetter eastern and drier western regions, AGB formed U‐shaped relationships with diversity indices (Simpson and Shannon–Wiener) in both areas (Figure 3). The *U*‐shape's asymmetry (steeper diversity decline in humid sites) reflects shifting community assembly mechanisms. In arid zones, biomass gains during diversity loss align with functional dominance; plant stress‐tolerant traits drive biomass via selection effects rather than niche complementarity (Aoyama et al. 2023). Conversely, in humid grasslands, high diversity initially supports complementary resource use. However, beyond a Shannon–Wiener index threshold (2.1), light competition favors dominance by tall, fast‐growing species that suppress neighbors (Jiang et al. 2022). This suggests grasslands increase productivity either by adding or losing species, depending on local conditions.

The composition of plant functional groups is intricately connected to the relationship between productivity and diversity in grassland plant species (Li et al. 2021). In our study area, the dominant plant species shifted from Chenopodiaceae to Gramineae as AGB increased in the eastern region. Conversely, the dominant family shifted from Chenopodiaceae to Compositae with an increasing diversity index in the western region (Figure S5). Across the sample zone, Gramineae has a higher water demand and is therefore more prevalent in the water‐rich eastern part. In contrast, Chenopodiaceae requires less water and is more prevalent in the transition zones between typical and desertified grasslands. Compositae is well‐adapted to arid environments and is typically found in arid desertified grasslands (Lv et al. 2021). The curve depicting the relationship between the diversity index and biomass suggests that the diversity threshold and changes in dominant species significantly improve productivity in degraded grasslands.

### 4.3 Linkage Between Plant Diversity and Spatial Dynamics of Soil Organic Carbon Decomposition

SOC mineralization directly influences soil carbon stocks and overall carbon balance (Zhang et al. 2024). In this study, MBC was identified as the key driver of potential cumulative mineralization of SOC (Figure 7). MBC was a stronger predictor of cumulative mineralization of SOC than SOC in alpine meadows (Shu et al. 2023). Previous controlled incubation experiments demonstrated that soil moisture dominantly regulates SOC mineralization by modulating microbial activity and SOC substrate accessibility (Mi et al. 2015). Most studies have identified microbial biomass as a crucial factor regulating organic carbon mineralization, thereby playing a key role (Dong et al. 2022).

In this study, both bacterial and fungal counts were positively correlated with cumulative carbon mineralization. However, the slope of fungal counts in relation to cumulative carbon mineralization of SOC was smaller than that of bacterial counts (Figure S4), suggesting that even small changes in bacterial counts can greatly influence carbon mineralization at different soil depths (0–5, 5–10, 10–20 cm). Consequently, bacteria contributed more to carbon mineralization than fungi (Ma et al. 2024), as bacterial communities are more specialized in decomposing labile carbon. In contrast, fungi play a more crucial role in breaking down more recalcitrant soil carbon (Myers et al. 2001). This suggested that soil bacterial flora dominates short‐term SOC decomposition. Although SOC quality was not chemically characterized, the disproportionate bacterial‐mineralization coupling (Figure S4) suggests labile C dominated short‐term decomposition. This aligns with bacterial dominance in labile C processing (Malik et al. 2020), and 28‐day incubations primarily reflecting rapid C pools (Wang et al. 2021). However, we must recognize the limitations in the process of integrating data. While air‐drying preserved sample comparability, it may alter microbial resuscitation dynamics (Powlson et al. 1987).

In the study of large‐scale spatial dynamics, SOC mineralization trends could often be inferred from the climate. SOC decomposition is especially responsive to temperature fluctuations (Curtin et al. 2012). However, our study found that increased MAT significantly reduced organic carbon mineralization in surface soils (0–10 cm) (*p* < 0.05) (Figure S2). In contrast, MAT had no detectable effect on subsoils (10–20 cm). Conversely, higher MAP strongly enhanced mineralization across all soil layers (*p* < 0.05). Higher MAT reduced surface soil moisture and suppressed microbial growth and activity, thereby decreasing organic carbon mineralization (Figure S4). SEM analysis demonstrated that the MinC was not directly tied to temperature but was indirectly influenced by precipitation (Figure 7), further confirming that precipitation exerts stronger control over SOC mineralization accumulation than temperature, highlighting the crucial regulatory role of precipitation in carbon cycling processes within temperate grassland ecosystems. Some studies have indicated that semi‐arid grasslands in Inner Mongolia are nitrogen‐limited (Gong et al. 2011). In our previous research, the fungal‐to‐bacterial ratio in eastern Inner Mongolia was shown to impact microbial carbon levels (Mi et al. 2022). Nitrogen influences soil microbial composition, and SOC influences cumulative carbon mineralized by MBC, though the mineralization process is affected by the gradient span (Wei et al. 2020).

We attempted to analyze the potential coupling between plant diversity and SOC sequestration. Our results showed that SOC mediated plant species diversity and organic carbon mineralization exhibited opposite trends (Figures 7 and S7). SOC influenced both plant species diversity and SOC mineralization by affecting soil nutrients and microbial biomass. Soil pH affects plant species diversity and SOC mineralization by altering soil nutrients and microbial communities (Chen et al. 2013). Enrichment of soil nutrients leads to acidification, and elevated pH levels exhibit a notable negative correlation with plant species diversity (Chen et al. 2022). In low‐diversity community, poor nutrient utilization drives plants to release labile root carbon, stimulating bacterial growth that accelerates soil organic matter decomposition to access nitrogen and phosphorus (Zhang et al. 2018).

Our results suggested that SOC and pH together control soil nutrient availability, with changes in these parameters altering microbial community structure. These changes further influence plant diversity and organic carbon mineralization rates. Soil nutrient–acidity interactions directly shape plant growth, species distribution, and adaptation. Enhanced biodiversity increased soil carbon input quality/quantity, promoting carbon sequestration and reducing ecosystem greenhouse gas emissions (Shrestha et al. 2018). Higher species richness reduced SOC‐derived CO_2_ emissions, demonstrating biodiversity‐driven suppression of mineralization. SOC and pH collaboratively affect soil nutrient levels, while changes in soil conditions result in alterations to microbial community structure and composition, which in turn influence plant species diversity and organic carbon mineralization. Thus, there is a synergistic relationship between SOC, pH, plant species diversity, and organic carbon mineralization.

A direct determinant of plant growth, including species distribution and adaptability, is the synergistic interaction of soil nutrients and acidity. Moreover, species diversity affects the quantity and quality of carbon inputs into the soil (Bello et al. 2015; Sobral et al. 2017). Higher biodiversity may lead to greater soil carbon sequestration and reduced greenhouse gas emissions from ecosystems (Shrestha et al. 2018; Losada et al. 2023). Consequently, local organic carbon mineralization can be assessed based on species richness in a community, and areas with higher species diversity are likely to release less CO_2_ from SOC mineralization. These findings enhance grassland biodiversity and stability by revealing climate–soil–microbe–biodiversity links in carbon cycling, thus aiding biodiversity predictions.

## 5 Conclusions

1. MAP primarily regulates community biomass in the semi‐arid steppe of southeastern Mongolia. The relationship between aboveground biomass and plant species diversity follows two strategies: higher and lower diversity with increased AGB. In the arid western region, diversity index thresholds were low (Shannon–Wiener index: 1.14, Simpson's index: 0.49). Conversely, in the humid eastern region, these thresholds were higher (Shannon–Wiener index: 2.19, Simpson's index: 0.72).
2. Plant diversity was directly driven by pH, SOC, and MAT, with decreases in these factors enhancing community plant diversity. This result suggests that shifts in climate and soil conditions could significantly impact species composition and ecosystem stability.
3. PG functional groups primarily dictate productivity. Conversely, PF functional groups serve as the key drivers of plant species diversity within these communities.
4. MBC is a primary driver of potential SOC mineralization, with MAP influencing MBC through its regulation of SOC. These findings emphasize the importance of microbial processes in carbon cycling under varying precipitation regimes.
5. The contrasting trends observed between plant species diversity and SOC mineralization are modulated by SOC. This suggests that organic carbon accumulation and plant community diversification may respond differently to environmental changes, with implications for carbon sequestration.

## Author Contributions

**Jia Mi:** conceptualization (equal), data curation (equal), methodology (equal), writing – original draft (equal). **Fei Wang:** writing – original draft (equal). **Jing Shi:** writing – review and editing (equal). **Qianju Wang:** data curation (equal). **Haiyan Pang:** data curation (equal). **Jianhao Yu:** investigation (equal). **Dima Chen:** writing – review and editing (equal). **Yongfei Bai:** conceptualization (equal), project administration (equal), supervision (equal), writing – review and editing (equal).

## Conflicts of Interest

The authors declare no conflicts of interest.

## Supporting information


Figures S1–S8.

## Data Availability

Data available via the Dryad Digital Repository https://doi.org/10.5061/dryad.9cnp5hqvp.
