# Supplementary material for: Contrasting Trends in Plant Diversity and Soil Carbon Mineralization Under Precipitation‐Driven Vegetation and Soil Carbon Dynamics in the Mongolian Plateau
Source: Ecol Evol. 2025 Jul 15;15(7):e71806. doi: 10.1002/ece3.71806 (PMC12263183; doi:10.1002/ece3.71806)
Supplement: Supplementary file 1 — Figures S1–S8. [file ECE3-15-e71806-s001.docx]

**Supporting Information**


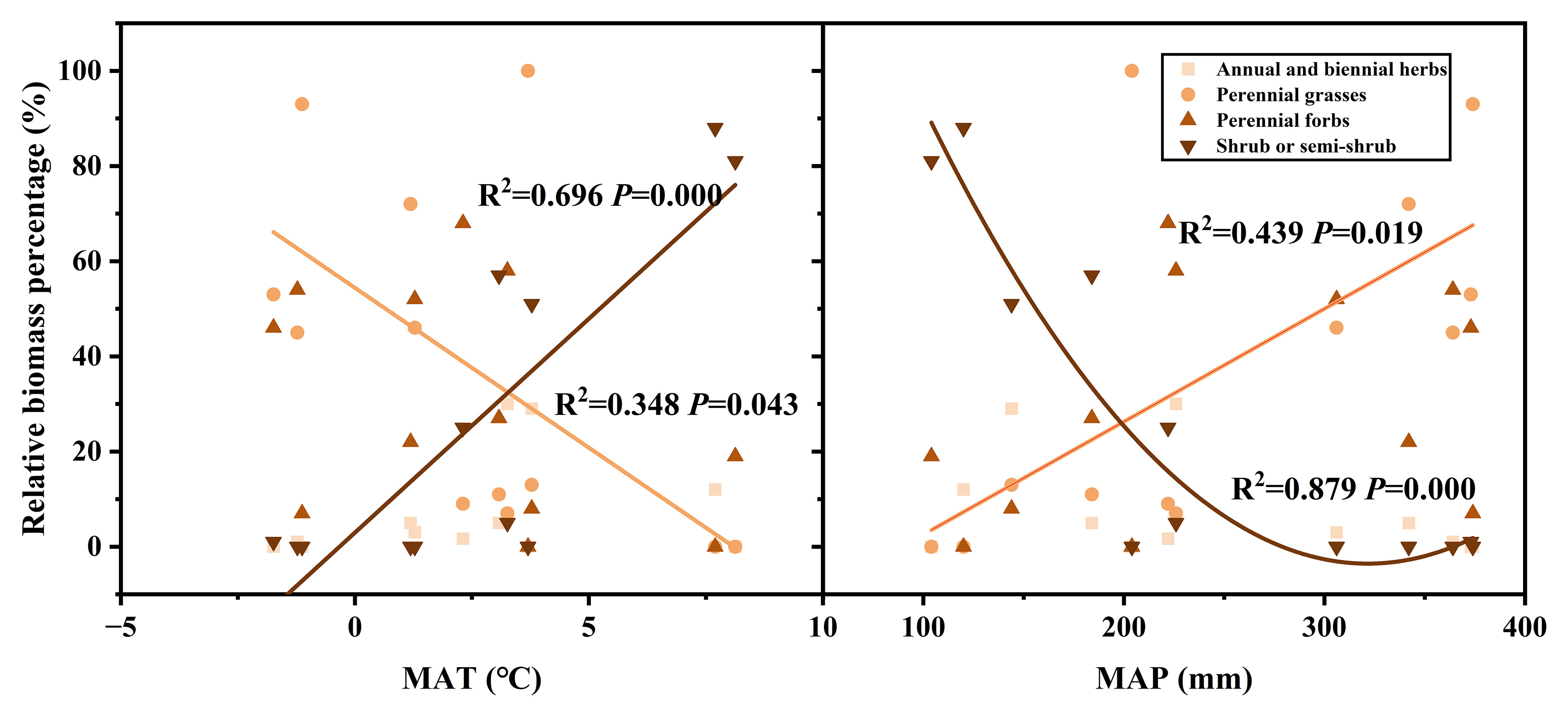


FIGURE S1 Relationship between mean annual temperature (MAT) and mean annual precipitation (MAP) and relative biomass of different plant functional groups.


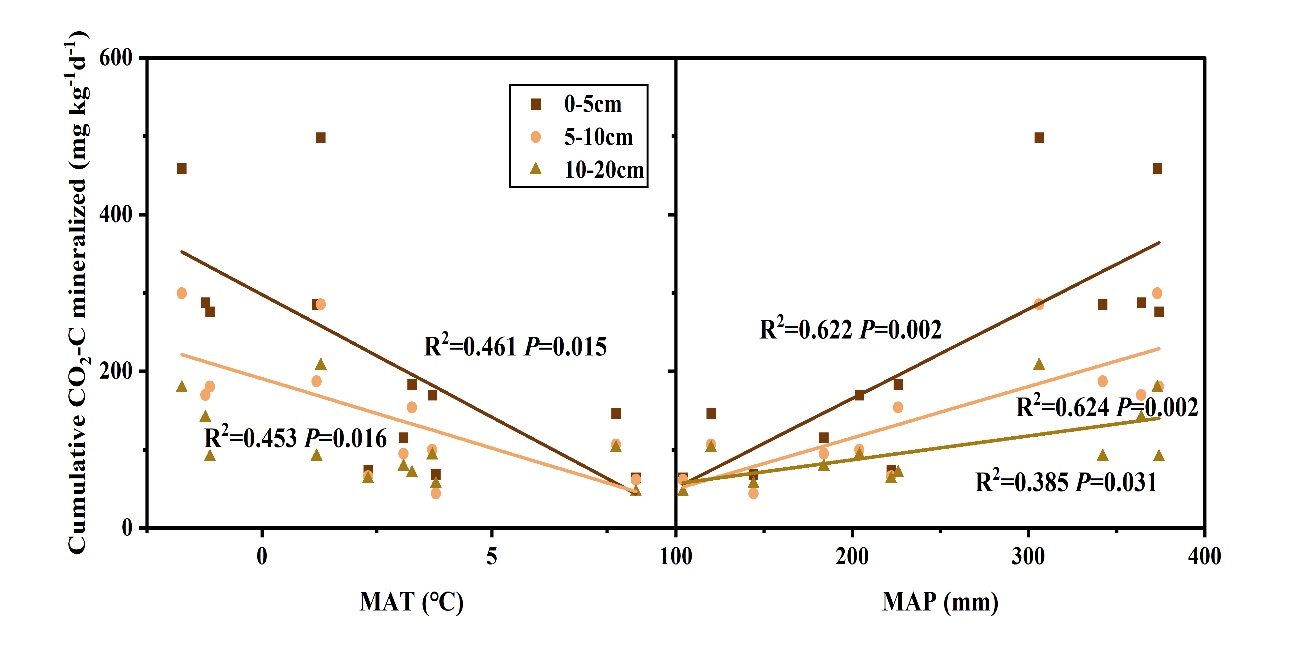


FIGURE S2 Relationship between mean annual temperature (MAT) and mean annual precipitation (MAP) and cumulative CO_2_-C from mineralised soil organic carbon


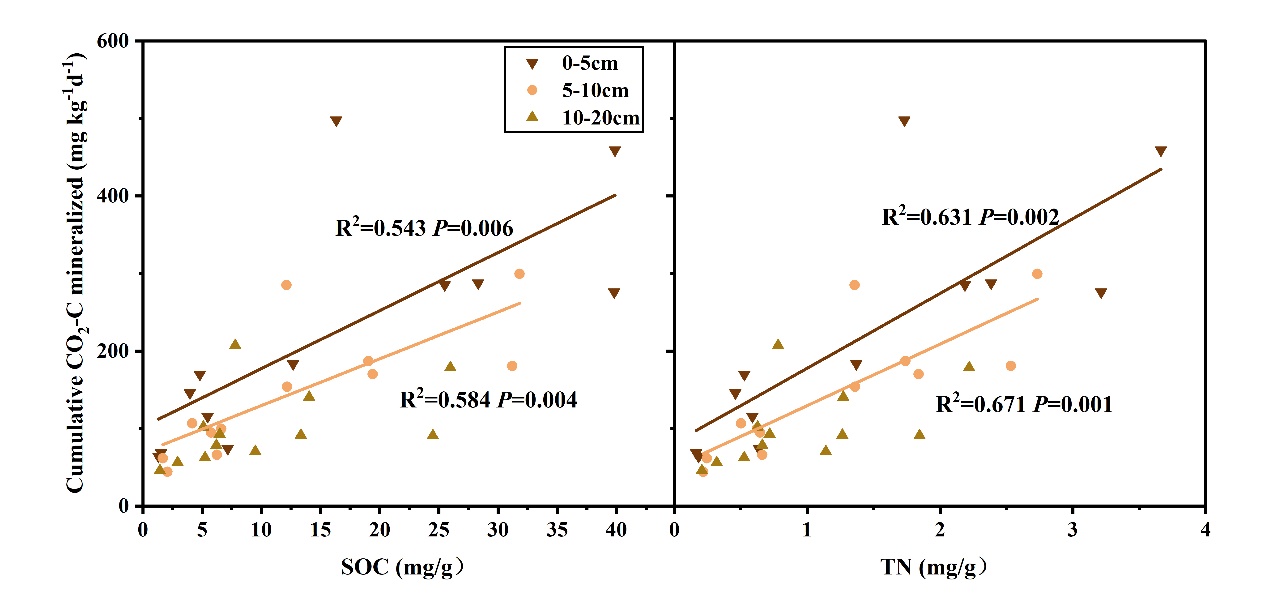


FIGURE S3 Relationship between soil organic carbon (SOC) and total nitrogen (TN) and cumulative CO_2_-C from mineralised SOC


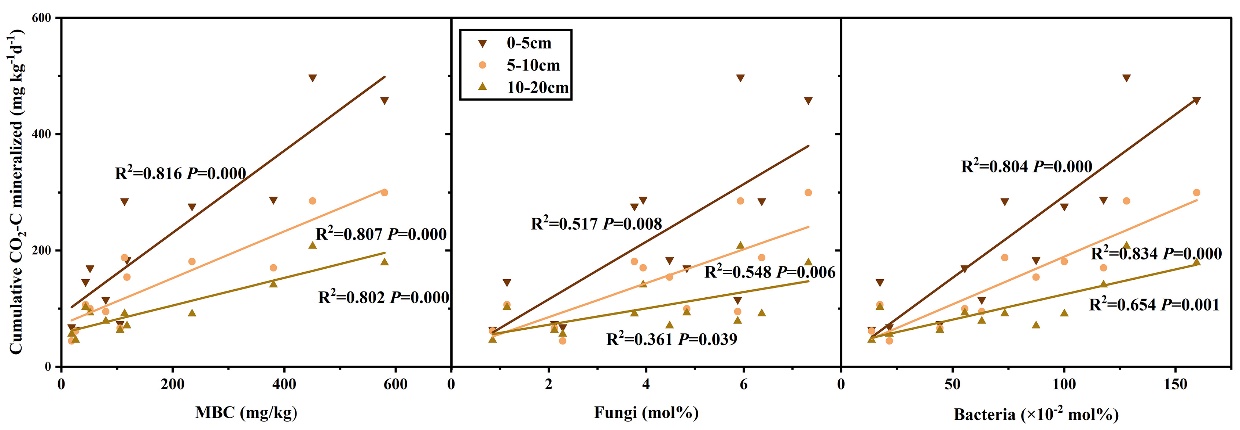


FIGURE S4 Relationship between MBC, fungi or bacteria counts and cumulative CO_2_-C from mineralised soil organic carbon


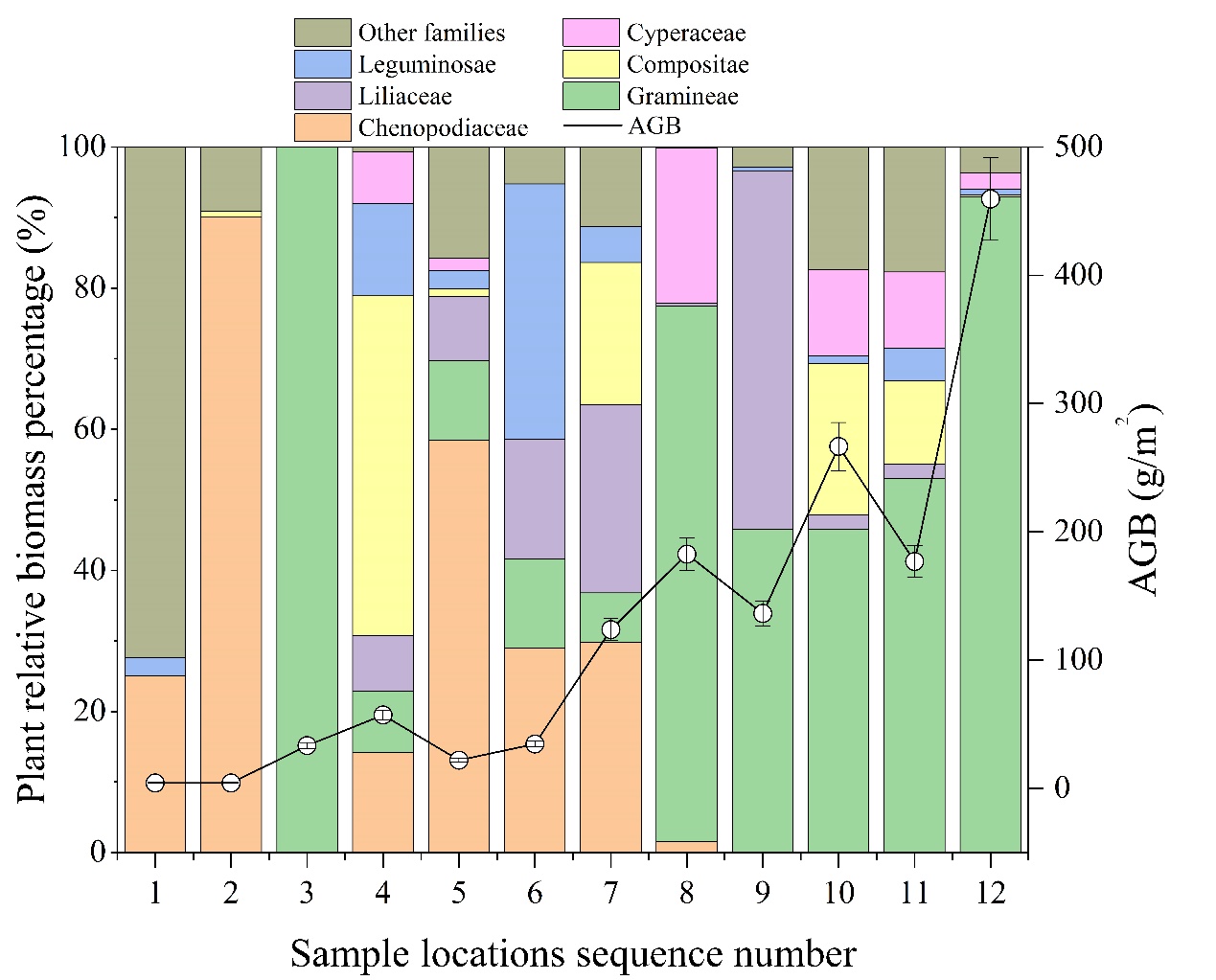


FIGURE S5 Aboveground biomass and biomass composition of plants of different families in the study area


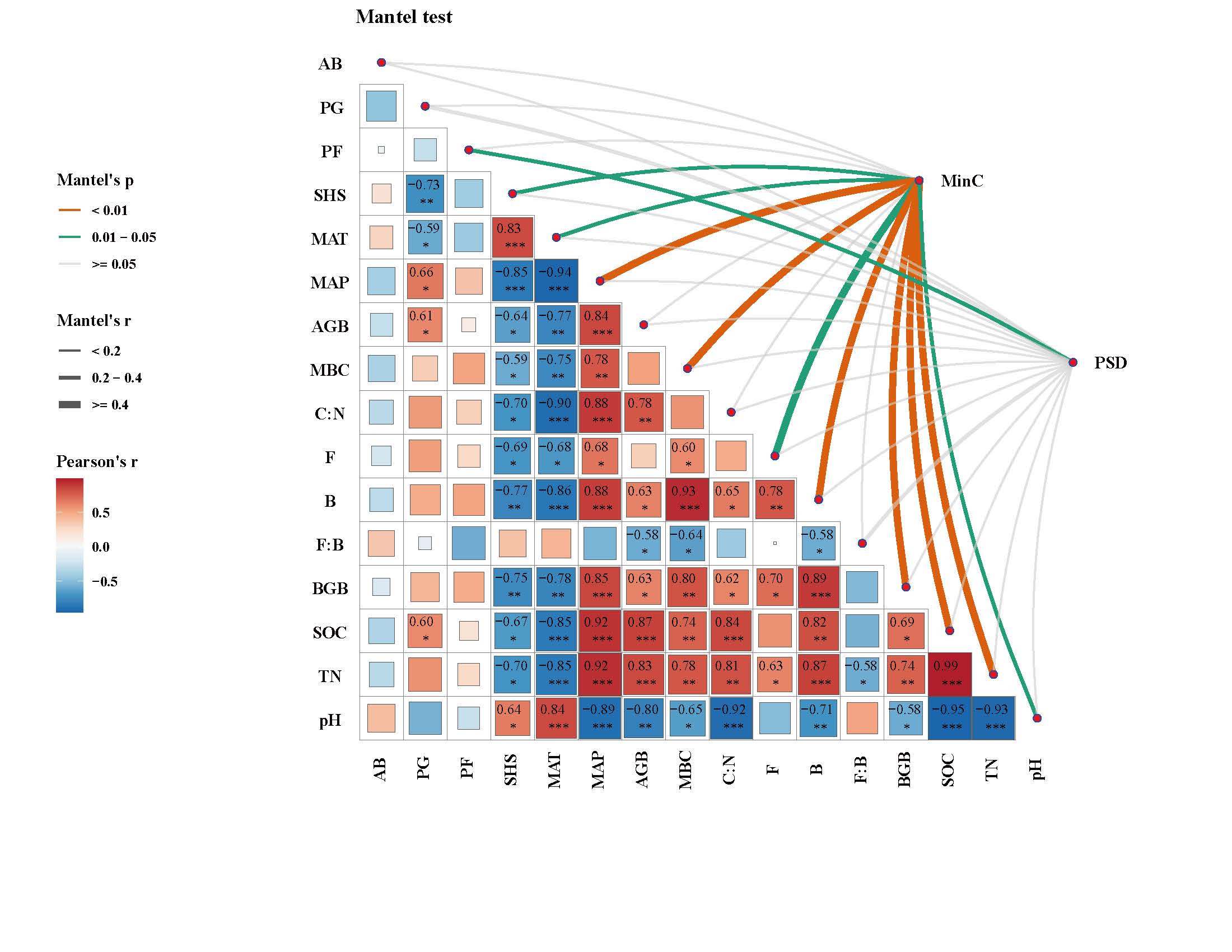


FIGURE S6 Correlation and Mantel test composite plots among climate, vegetation, and soil factors. The color indicates the Pearson’s correlation coefficient. The width and color of the lines represent the statistic and significance p-value of the correlation coefficient r, respectively (significance is marked **P*<0.05, ***P*<0.01, and ****P*<0.001). AB: annual and biennial herbs; PG: perennial grasses; PF: perennial forbs; SHS: shrubs or subshrub plants; MAT, mean annual air temperature; MAP, mean annual precipitation; pH, soil pH value; SOC, soil organic carbon; MBC, soil microbial biomass carbon; MinC: cumulative mineralization of SOC; PSD, community species diversity (Shannon-Wiener diversity index was used in this model); C:N, soil carbon to nitrogen ratio; TN, total soil nitrogen; AGB, above-ground biomass; BGB, below-ground biomass; F:B, soil fungi and bacteria ratio; F: Fungal count of soil; B: Bacterial count of soil.


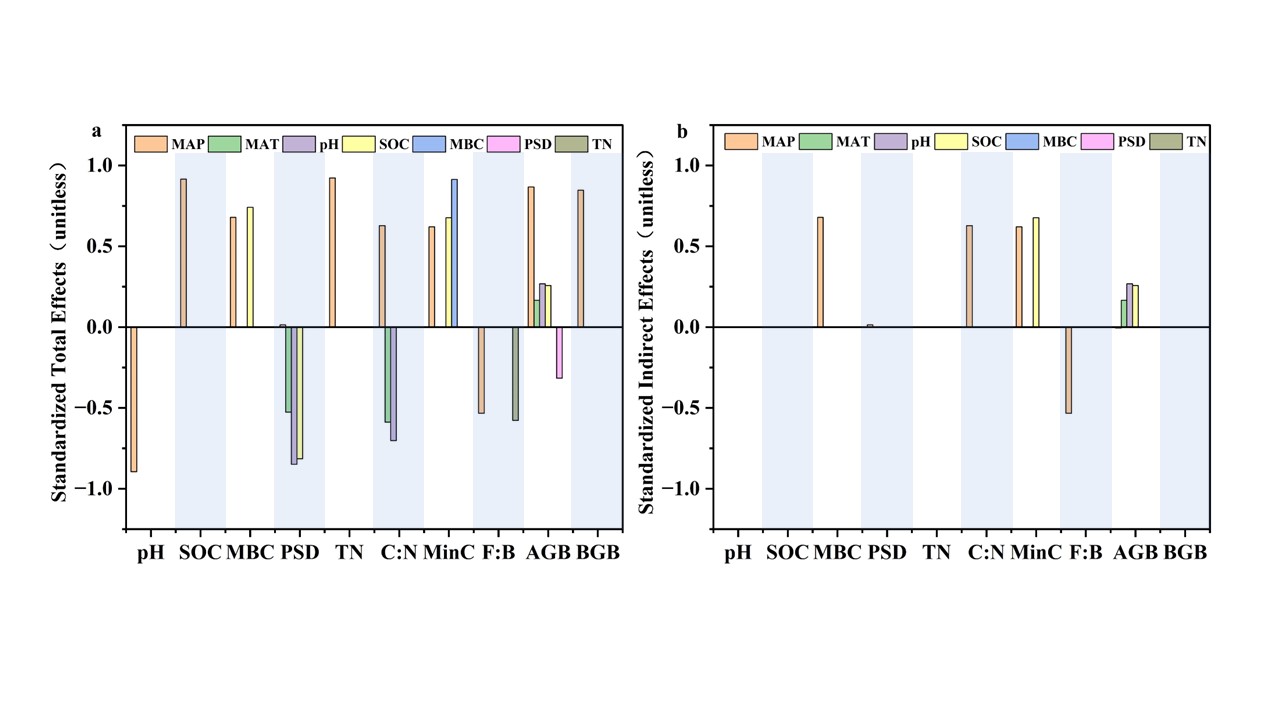


FIGURE S7 Effects of climate and soil factors on plant and microbial factors fitted to standardized total effects (a) and indirect effects (b) analyses based on structural equation modeling (SEM). MAP, mean annual precipitation; MAT, mean annual air temperature; pH, soil pH value; SOC, soil organic carbon; MBC, soil microbial biomass carbon; PSD, community species diversity (Shannon-Wiener diversity index was used in this model); C:N, soil carbon to nitrogen ratio; TN, total soil nitrogen; AGB, above-ground biomass; BGB, below-ground biomass; F:B, soil fungi and bacteria ratio; MinC: cumulative mineralization of SOC.


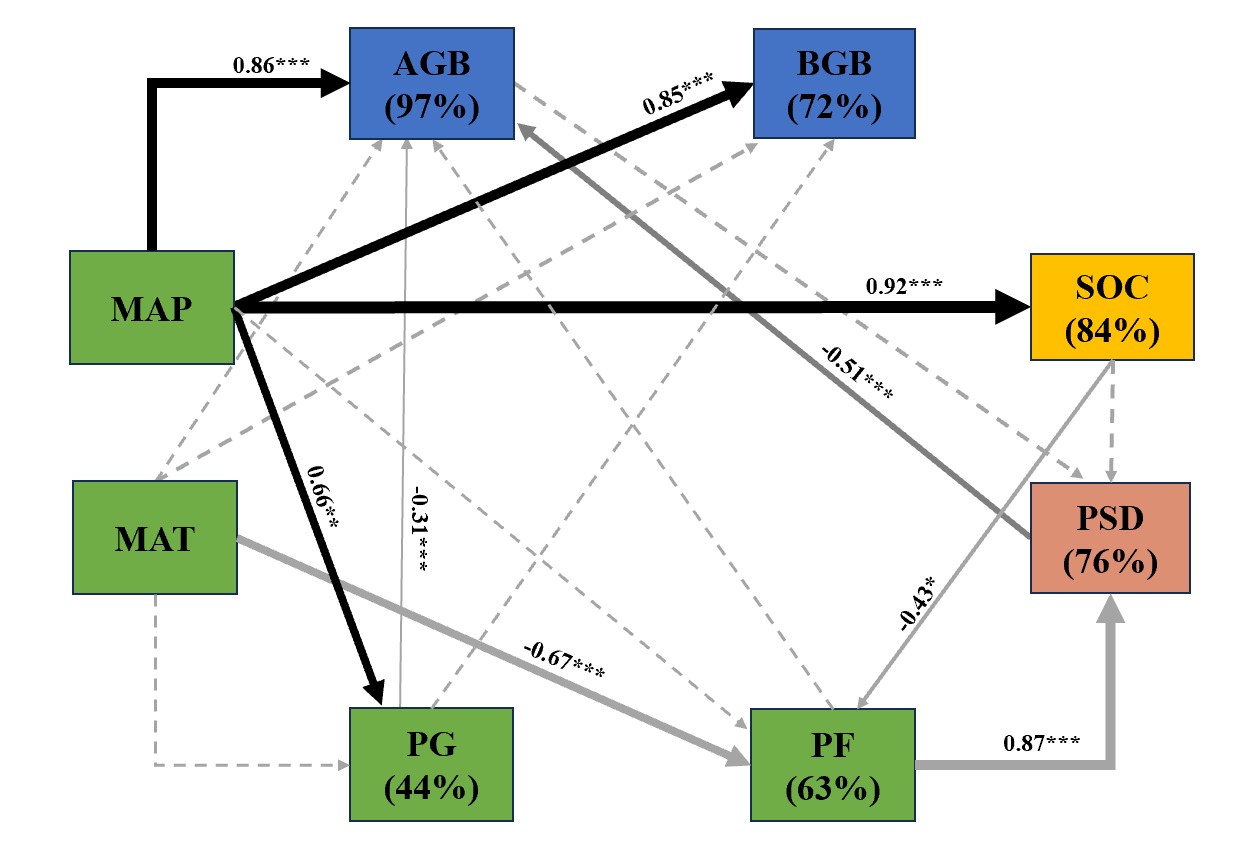


Figure S8 The influence pathway of climate, biomass, functional groups, soil factors, and plant diversity fitted by SEM analysis. SEM analysis showed a good fit of the hypothesised model (*χ*^2^ = 104.423, d*f* = 26, *P* = 0.000). Black arrows indicate positive correlations, while gray arrows indicate negative correlations. MAP, mean annual precipitation; MAT, mean annual air temperature; SOC, soil organic carbon; PSD, community species diversity (Shannon-Wiener diversity index was used in this model); AGB, aboveground biomass; BGB, belowground biomass; PG, perennial grasses; PF, perennial forbs
